# Role of lysophosphatidic acid receptor LPA_2 _in the development of allergic airway inflammation in a murine model of asthma

**DOI:** 10.1186/1465-9921-10-114

**Published:** 2009-11-20

**Authors:** Yutong Zhao, Jiankun Tong, Donghong He, Srikanth Pendyala, Berdyshev Evgeny, Jerold Chun, Anne I Sperling, Viswanathan Natarajan

**Affiliations:** 1 Department of Medicine, The University of Chicago, Chicago, Illinois, USA; 2 Department of Molecular Biology, The Scripps Research Institute, La Jolla, California, USA

## Abstract

**Background:**

Lysophosphatidic acid (LPA) plays a critical role in airway inflammation through G protein-coupled LPA receptors (LPA_1-3_). We have demonstrated that LPA induced cytokine and lipid mediator release in human bronchial epithelial cells. Here we provide evidence for the role of LPA and LPA receptors in Th2-dominant airway inflammation.

**Methods:**

Wild type, LPA_1 _heterozygous knockout mice (LPA_1_^+/-^), and LPA_2 _heterozygous knockout mice (LPA_2_^+/-^) were sensitized with inactivated *Schistosoma mansoni *eggs and local antigenic challenge with *Schistosoma mansoni *soluble egg Ag (SEA) in the lungs. Bronchoalveolar larvage (BAL) fluids and lung tissues were collected for analysis of inflammatory responses. Further, tracheal epithelial cells were isolated and challenged with LPA.

**Results:**

BAL fluids from *Schistosoma mansoni *egg-sensitized and challenged wild type mice (4 days of challenge) showed increase of LPA level (~2.8 fold), compared to control mice. LPA_2_^+/- ^mice, but not LPA_1_^+/- ^mice, exposed to *Schistosoma mansoni *egg revealed significantly reduced cell numbers and eosinophils in BAL fluids, compared to challenged wild type mice. Both LPA_2_^+/- ^and LPA_1_^+/- ^mice showed decreases in bronchial goblet cells. LPA_2_^+/- ^mice, but not LPA_1_^+/- ^mice showed the decreases in prostaglandin E2 (PGE2) and LPA levels in BAL fluids after SEA challenge. The PGE2 production by LPA was reduced in isolated tracheal epithelial cells from LPA_2_^+/- ^mice. These results suggest that LPA and LPA receptors are involved in *Schistosoma mansoni *egg-mediated inflammation and further studies are proposed to understand the role of LPA and LPA receptors in the inflammatory process.

## Background

Lysophosphatidic acid (LPA) is a naturally occurring bioactive lysophospholipid and is a component of plasma, biological fluids, and tissues [1-3]. Many of the biological responses of LPA such as cell proliferation [4,5], migration [6,7], and cytokine release [8-10] are mediated by a family of G-protein coupled receptors (GPCRs). At least six LPA receptors, LPA_1-6_, have been cloned and described in mammals, and the biological effects of LPA are mediated by ligation to specific LPA receptors that are coupled to heterotrimeric G-protein families, the G_s_, G_i_, G_q_, and G_12/13 _[11-17].

The role of LPA and LPA receptors in airway inflammatory diseases have been studied *in vitro *and *in vivo*. LPA is a potent stimulator of interleukin-8 (IL-8) secretion in primary cultured human bronchial epithelial cells (HBEpCs) [8,10], and is a mitogen for airway smooth muscle cells [18,19]. Intratracheal administration of LPA in mice increased MIP-2 levels at 3 h and neutrophil infiltration at 6 h [20]. Inhalation of LPA induced histamine release and enhanced the recruitment eosinophils and neutrophils to the guinea pig lung alveolar space [21,22]. While these studies suggest that LPA regulates airway inflammation via stimulating the release of cytokines and inflammatory mediators that modulate infiltration of neutrophils and eosinophils into the airway, others point out that LPA exhibits anti-inflammatory effects and promotes resolution of inflammation. In human bronchial epithelial cells, LPA induced IL-13 decoy receptor, IL-13Rα2 expression and release, and attenuated IL-13-induced phosphorylation of STAT6 [9]. Further, LPA enhanced cyclooxygenase-2 (COX-2) expression and prostaglandin E2 (PGE2) release in HBEpCs [23] suggesting a protective role in the innate immunity response and tissue repair process in airway inflammation [24,25]. Recently, Fan et al. showed that intravenous injection with LPA attenuated bacterial endotoxin-induced plasma TNF-α production and myeloperoxidase activity in mouse lung, suggesting an anti-inflammatory role of LPA in a murine model of acute lung injury [26]. In addition to its anti-inflammatory effect, LPA regulated E-cadherin intracellular trafficking and airway epithelial barrier integrity and intratracheal post-treatment with LPA reduced neutrophil influx, protein leak, and E-cadherin shedding in bronchoalveolar lavage (BAL) fluids in a murine model of LPS-induced acute lung injury [27]. These data suggest a protective role of administrated LPA in airway inflammatory diseases.

In contrast to several *in vitro *studies on the role of LPA as a pro- or anti-inflammatory mediator in airway epithelial and smooth muscle cells [8,10,18-20], there are a few reports linking LPA levels and LPA receptors to airway or lung inflammation and injury. We have recently shown that LPA was constitutively present in BAL fluids from normal and asthmatic subjects and segmental allergen challenge increased LPA levels in BAL fluids significantly [28]. However, the source of LPA and the pathophysiological relevance of increased LPA after segmental allergen challenge to allergic inflammation remain to be elucidated. Similarly, LPA levels in BAL fluids from individuals with idiopathic pulmonary fibrosis were significantly higher compared to normal controls [29]. Further, an increase in LPA levels in BAL fluid following lung injury was observed in the bleomycin model of pulmonary fibrosis, and mice lacking LPA_1 _were protected from fibrosis and mortality [29]. These studies suggest a role for LPA receptors in linking lung injury in the murine bleomycin model of pulmonary fibrosis.

Asthma is a chronic inflammatory disease of the airways involving T-lymphocytes and eosinophils infiltration, mucus overproduction and airway hyper-responsiveness. Inflammatory mediators including lipid mediators play a critical role in the pathogenesis of chronic airway diseases and facilitate the recruitment, activation, and trafficking of inflammatory cells in the airways. Very little is known on the physiological consequences of increased LPA levels and role of LPA receptors in asthma. To address the role of LPA receptors in Th2-mediated inflammation, we have used a well described *Schistosoma mansoni *eggs-sensitized murine model of allergic airway inflammation [30-32]. Control wild type, *LPA*_1_^+/- ^and *LPA*_2_^+/- ^mice were sensitized and challenged with *Schistosoma mansoni *eggs. LPA_2_^+/- ^challenged mice compared to wild type showed decrease in cell numbers, eosinophils, and positive PAS staining. Interestingly, only *Schistosoma mansoni *eggs sensitized and challenged *LPA*_2_^+/-^, but not *LPA*_1_^+/-^, mice showed reduced PGE2 levels in BAL fluids which correlated with diminished COX-2 expression in LPA_2_^+/- ^mice. Furthermore, airway epithelial cells isolated from *LPA*_2_^+/- ^mice exhibited reduced COX-2 expression and PGE2 release compared to cells from wild type mice. These results show for the first time a role for LPA_2 _in the development of airway inflammation and pathogenesis of asthma.

## Materials and methods

### Animals

All the mice were bred and housed in a specific pathogen-free barrier facility maintained by the University of Chicago Animal Resources Center. The studies reported here conform to the principles outlined by the Animal Welfare Act and the National Institutes of Health guidelines for the care and use of animals in biomedical research.

### PCR genotyping of LPA_1_^+/- ^and LPA_2_^+/- ^mice

Extract-N-Amp Tissue PCR kit (Sigma Aldrich, S. Louis) was utilized for isolating genomic DNA from mouse tail and amplifying DNA fragments. The primers for LPA_1 _and LPA_2 _knockout mice were described as previous studies [33,34].

### *Schistosoma mansoni *eggs sensitization and challenge

*Schistosoma mansoni *eggs sensitization and challenge to induce murine allergic airway disease were described before [31]. In brief, at day 0, mice (6-8 weeks) were immunized by i.p. injection of 5,000 inactivated *Schistosoma mansoni *eggs. At day 7, the mice were challenged with 10 μg of SEA by intratracheal aspiration. The mice were studied at day 11.

### Analyses of BAL fluids

BAL fluids were performed by an intratracheal injection of 1 ml of PBS solution followed by gentle aspiration. The lavage was repeated twice to recover a total volume of 1.8-2.0 ml. The lavage was centrifuged and supernatant was processed for PGE2 or LPA measurement. The percentages of cell types in BAL fluids were determined by FACS analysis with cell type-specific markers.

### Histology

Lungs were removed from mice and lobes were sectioned sagitally, embedded in paraffin, cut into 5-μm sections. Periodic Acid Schiff (PAS) staining were performed by Pathology Core Facility in The University of Chicago.

### Antibodies and flow cytometry

Antibody to mouse CCR3 (clone 831101.111) was obtained from R&D Systems (Minneapolis, MN). Cells were fixed with 4% paraformaldehyde for 10 min and incubated with staining antibodies for 30 min at 4°C. The samples were washed and analyzed on a FACS LSR-II (Becton Dickinson).

### Isolation of tracheal epithelial cells

Briefly, mice were euthanized and their tracheas were isolated and digested with 0.1% protease (Type XIV, Sigma) overnight at 4°C. The tracheal cell suspension were transferred to 15 ml tube and spun at 1500 rpm for 3 min at 4°C and were pooled in BEGM medium (Lonza, Walkersville, MD).

### LPA measurement by mass spectrometry

Lipids in BAL were extracted as described before [28]. In brief, LPA levels were determined using liquid chromatography and tandem mass spectrometry (LC) with ABI-4000 Q-TRAP hybrid triple quadrupole/ion trap mass spectrometer (MS) coupled with an Agilent 1100 liquid chromatography system. Lipids were separated using methanol/water/HCOOH, 79/20/0.5, v/v, with 5 mM NH4COOH as solvent A and methanol/acetonitrile/HCOOH, 59/40/0.5, v/v, with 5 mM NH4COOH as Solvent B. LPA molecular species were analyzed in negative ionization mode with declustering potential and collision energy optimized for LPA.

### PGE2 measurement

Mouse tracheal epithelial cells grown on 6-well plates were challenged with LPA for 3 h, medium were collected and centrifuged at 5,000 × g for 10 min at 4°C. The supernatant or BAL fluid supernatant were transferred to new 2.0 ml-eppendorf tubes and frozen in -80°C for later analysis. Measurement of PGE2 levels, as 13, 14-dihydro-15-keto PGE2, was carried out using a commercial ELISA kit according to manufacture's instruction.

### RNA isolation and Real-time RT-PCR

Total RNA was isolated from cultured mouse tracheal epithelial cells using TRIzol^® ^reagent (Life Technology, Rockville, MD) according to the manufacturer's instructions. RNA was quantified spectrophotometrically and 1 μg of RNA was reversed transcripted using cDNA synthesis kit (Bio-Rad) and Real-time PCR and quantitative PCR were performed to assess expression of the COX-2, LPA_1_, LPA_2_, LPA_3_, LPA_4_, and LPA_5 _using primers designed based on mouse mRNA sequences (Table 1.). Amplicon expression in each sample was normalized to its 18S RNA content. The relative abundance of target mRNA in each sample was calculated as 2 raised to the negative of its threshold cycle value times 10^6 ^after being normalized to the abundance of its corresponding 18S, [e.g., 2 ^-(Target Gene Threshold Cycle)^/2 ^-(18S Threshold Cycle) ^× 10^6^].

**Table 1:** Primers for mouse LPA receptors and COX-2

LPA_1_	Forward: 5'-TCAACCTGGTGACCTTTGTG-3' Reverse: 5'-GGTCCAGAACTATGCCGAGA-3'
LPA_2_	Forward: 5'-ATATTCCTGCCGAGATGCTG-3' Reverse: 5'-AAGCTGAGTAACGGGCAGAC-3'

LPA_3_	Forward: 5'-ATTGCCTCTGCAACATCTCG-3' Reverse: 5'-ATGAAGAAGGCCAGGAGGTT-3'

LPA_4_	Forward: 5'-ACTGCGTTCCTCACCAACAT-3' Reverse: 5'-CGATCGGAAGGGATAGACAA-3'

LPA_5_	Forward: 5'-GCTCCAGTGCCCTGACTATC-3' Reverse: 5'-CAGAGCGTTGAGAGGGAGAC-3'

COX-2	Forward: 5'-CCCCCACAGTCAAAGACACT-3' Reverse: 5'-GGCACCAGACCAAAGACTTC-3'

### Western blotting

Equal amounts of protein (20 μg) were subjected to 10% SDS/PAGE gels, transferred to polyvinylidene difluoride membranes, blocked with 5% (w/v) BSA in TBST (25 mM Tris-HCl, pH 7.4, 137 mM NaCl and 0.1% Tween-20) for 1 h and incubated with anti-COX-2 antibody in 5% (w/v) BSA in TBST for 1-2 h at room temperature. The membranes were washed at least three times with TBST at 15 min intervals and then incubated with a rabbit horseradish peroxidase-conjugated secondary antibody (1: 3,000) for 1 h at room temperature. The membrane was developed with enhanced chemiluminescence detection system according to Manufacturer's instructions.

### Statistical analysis

All results were subjected to statistical analysis using one-way ANOVA and, whenever appropriate, analyzed by Student-Newman-Keuls test. Data are expressed as means ± S.D. of triplicate samples from at least three independent experiments and level of significance was taken to P < 0.05.

## Results

### *Schistosoma mansoni *eggs sensitization and challenge increases LPA levels in BAL fluids

**Figure 1:**
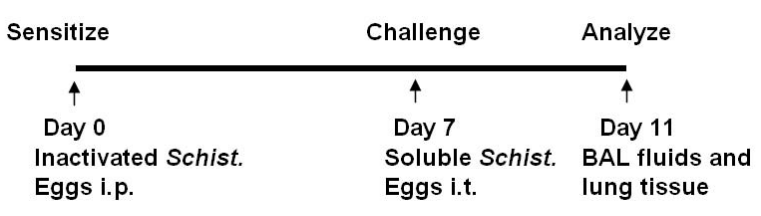
***Schistosoma mansoni *eggs sensitization and challenge induces murine asthmatic model**. At day 0, mice (6-8 weeks) were immunized by i.p. injection of 5,000 inactivated *Schistosoma mansoni *eggs. At day 7, mice were challenged with 10 μg of SEA by intratracheal aspiration. Lung tissues and BAL fluids were collected at day 11.

To investigate the role of LPA receptors in pathogenesis of asthma, we quantified LPA levels in BAL fluids from control and SEA-challenged mice. Mice were sensitized by i.p. injection of 5,000 inactivated *Schistosoma mansoni *eggs. At day 7, mice were challenged with or without 10 μg of SEA by intratracheal aspiration and at day 11, BAL fluids were collected (Fig. 1) and lipid were extracted and LPA levels in BAL fluids were measured by LC-MS/MS with C17:0 LPA as an internal standard. As shown in Table 2, LPA was detectable (~1254.3 ± 357.0 pmole/ml) in control mice (sensitized with inactivated *Schistosoma mansoni *eggs but not SEA challenged), and there was a ~2.8 fold increase in LPA levels (~3557.9 ± 109.3 pmole/ml) in *Schistosoma mansoni *eggs sensitized and challenged mice, compared to control mice. Unsaturated molecular species of LPA (18:1, 20:4, 22:5, and 22:6) were detected in BAL fluids of control mice, which increased significantly after SEA challenge. These results show for the first time, to our knowledge, increase in LPA during allergic lung inflammation in a murine model of asthma.

**Table 2:** Quatification of LPA molecular species in BAL fluids

LPA molecular species	Wt sensitization only (pmol/ml)	Wt sensitization and SEA challenged (pmol/ml)
14:0-LPA	5.6 ± 3.1	14.6 ± 1.2

16:1-LPA	50.4 ± 37.0	160.9 ± 3.7

16:0-LPA	88.4 ± 51.7	253.6 ± 10.8

18:2-LPA	83.9 ± 54.6	274.4 ± 7.0

18:1-LPA	154.3 ± 114.7	517.6 ± 17.9

18:0-LPA	54.9 ± 27.4	153.7 ± 8.0

20:5-LPA	26.7 ± 22.0	95.7 ± 1.9

20:4-LPA	212.9 ± 168.2	739.0 ± 23.1

20:3-LPA	51.7 ± 39.8	165.2 ± 5.5

20:2-LPA	4.7 ± 3.4	14.1 ± 0.8

22:6-LPA	189.4 ± 114.4	548.9 ± 18.1

22:5-LPA	291.6 ± 166.2	576.9 ± 22.2

22:4-LPA	16.8 ± 9.5	41.1 ± 1.5

22:3-LPA	0.7 ± 0.6	1.9 ± 0.5

22:2-LPA	0.2 ± 0.1	0.2 ± 0.1

Total LPA	1254.3 ± 357.0	3557.9 ± 109.3

BAL fluids were collected and lipis were extracted. LPA molecular species were quantified by LC-MS/MS with 17:0LPA as standard.

### *Schistosoma mansoni *eggs sensitization and challenge-induced airway inflammation is dependent on LPA_1 _and LPA_2_

**Figure 2:**
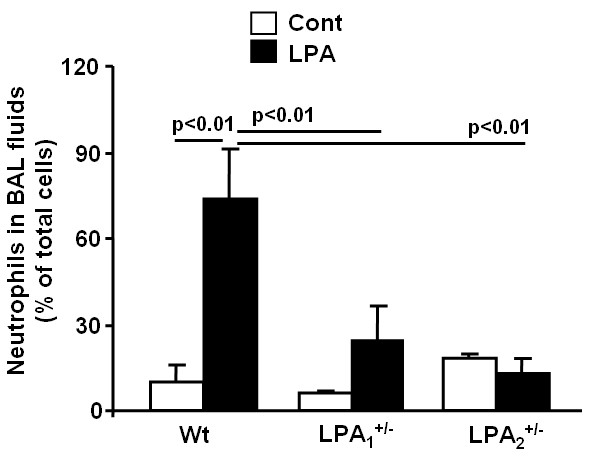
**LPA_1_^+/- ^and LPA_2_^+/- ^mice show reduced neutrophils infiltration to BAL fluids**. 18:1LPA (5 μM in 25 μl PBS) were intratracheally injected to wild type, LPA_1_^+/-^, and LPA_2_^+/- ^mice (n = 4-5) for 6 h. BAL fluids were collected and percentage of neutrophils in total cells were examined by Cytospin.

To determine the role of LPA receptors in airway inflammation mediated by *Schistosoma mansoni *eggs sensitization and challenge, we used LPA_1 _and LPA_2 _deficient mice, which were genetically engineered as described earlier [33,34]. The heterozygous LPA_1_^+/- ^and LPA_2_^+/- ^mice were housed and bred at the University of Chicago Animal Resources Center and described experiments were approved by the ACIU of the University of Chicago. Genotyping analyses with specific primers confirmed generation of wild type (+/+), heterozygous (+/-) and homozygous mice (-/-) from the genetically engineered LPA_1 _and LPA_2 _mice (data not shown). Since LPA_1_^-/- ^showed 50% neonatal lethality and impaired sucking in neonatal pups, all experiments were carried out with LPA_1_^+/- ^and LPA_2_^+/- ^mice to investigate the role of LPA receptors in *Schistosoma mansoni *eggs sensitization and challenge-mediated allergic inflammatory responses. To determine whether LPA_1_^+/- ^and LPA_2_^+/- ^mice reduced the effect of LPA, wild type, LPA_1_^+/- ^and LPA_2_^+/- ^mice were intratracheal challenged with 18:1LPA (5 μM in 25 μl PBS) for 6 h. As shown in Fig. 2, LPA challenge increased neutrophil infiltration, however, LPA_1_^+/- ^and LPA_2_^+/- ^mice reduced LPA-induced neutrophil infiltration in BAL fluids, suggesting that less LPA_1 _and LPA_2 _receptors in LPA_1_^+/- ^and LPA_2_^+/- ^mice reduce LPA-induced inflammation in lung and that LPA_1_^+/- ^and LPA_2_^+/- ^mice are useful models for investigating role of LPA receptors in lung inflammatory diseases.

**Figure 3:**
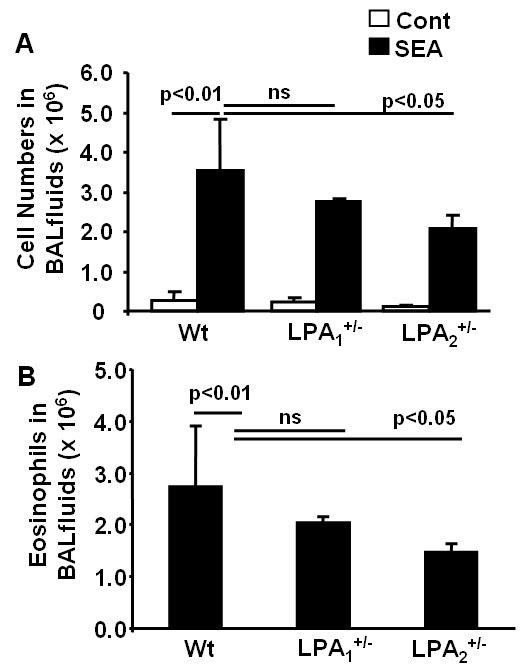
**LPA_2_^+/- ^mice exhibit a decrease in cell numbers and eosinophils in BAL fluids**. After wild type, LPA_1_^+/-^, and LPA_2_^+/- ^mice (n = 4-6) were challenged with or without *Schistosoma mansoni *eggs at day 11, as described in Materials and Methods, BAL fluids were collected and total cell numbers were accounted **(A)**. Eosinophil numbers were examined by flow cytometry with antibody to CCR3 **(B)**.

Wild type, LPA_1_^+/-^, and LPA_2_^+/- ^mice were sensitized with inactivated *Schistosoma mansoni *eggs and challenged with or without SEA for 4 days, BAL fluids and lung tissues were collected, cell numbers were measured under microscope and total eosinophils were determined by flow cytometry using eosinophils specific antibody (anti-CCR3). Consistent with pervious reports [30,32], *Schistosoma mansoni *eggs sensitized and challenged wild type mice showed significant increase in total cell numbers and eosinophils in BAL fluids; however, total cell numbers and recruitment of eosinophils were attenuated in LPA_2_^+/-^, but not LPA_1_^+/- ^mice (Fig. 3). These results suggest a role for LPA_2 _in influx of eosinophils into alveolar space during allergic inflammatory response.

**Figure 4:**
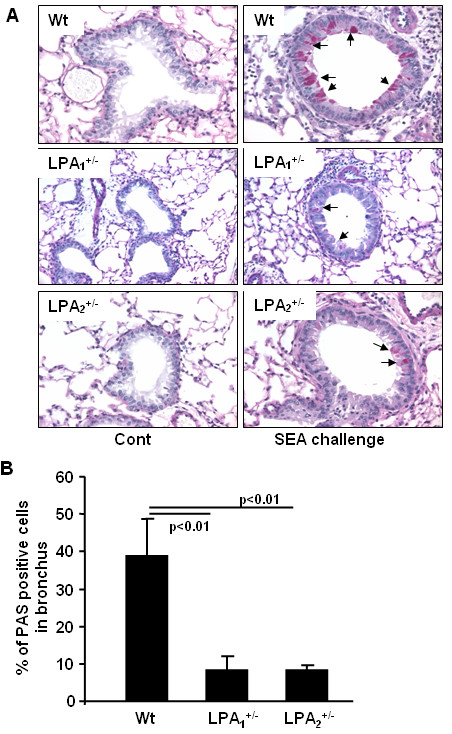
**LPA_1_^+/- ^and LPA_2_^+/- ^mice exhibit decreases in goblet cells**. **A) **Representative PAS staining sections from *Schistosoma mansoni *eggs unchallenged and challenged wild type, LPA_1_^+/-^, and LPA_2_^+/- ^mice (n = 4-6) are shown. **B) **Percentage of PAS positive goblet cells in each bronchia (n = 3-5) were calculated.

Airway goblet cell metaplasia and mucus production, indices of degree of inflammation, are hallmarks of asthma. Goblet cell metaplasia and mucus production were determined by PAS staining of histological sections of lung tissues from *Schistosoma mansoni *eggs sensitized and challenged or non-challenged wild type, LPA_1_^+/-^, and LPA_2_^+/- ^mice. As shown in Fig. 4A, PAS positive goblet cells were higher in *Schistosoma mansoni *eggs sensitized and challenged wild type mice, compared to *Schistosoma mansoni *eggs sensitized and non-challenged wild type mice (control mice), whereas significantly less PAS stained goblet cells were seen in *Schistosoma mansoni *eggs sensitized and challenged LPA_1_^+/- ^and LPA_2_^+/- ^mice, compared to *Schistosoma mansoni *eggs sensitized and challenged wild type mice. Scoring of the histological sections also confirmed a significantly higher percentage of bronchi for PAS positive stained cells in the sensitized and challenged control wild type mice compared to LPA_1_^+/- ^and LPA_2_^+/- ^mice (Fig. 4B). These results demonstrate that *Schistosoma mansoni *eggs sensitized and challenged LPA_1_^+/- ^and LPA_2_^+/- ^mice develop reduced goblet cell metaplasia and mucus production compared to control wild type mice. Together, these data suggest a role for LPA receptors for optimal induction of Th2-mediated airway inflammation.

### LPA_2_^+/-^, but not LPA_1_^+/-^, mice exhibit reduced LPA and PGE2 levels in BAL fluids, and COX-2 expression in lungs of *Schistosoma mansoni *eggs sensitized/challenged mice

**Figure 5:**
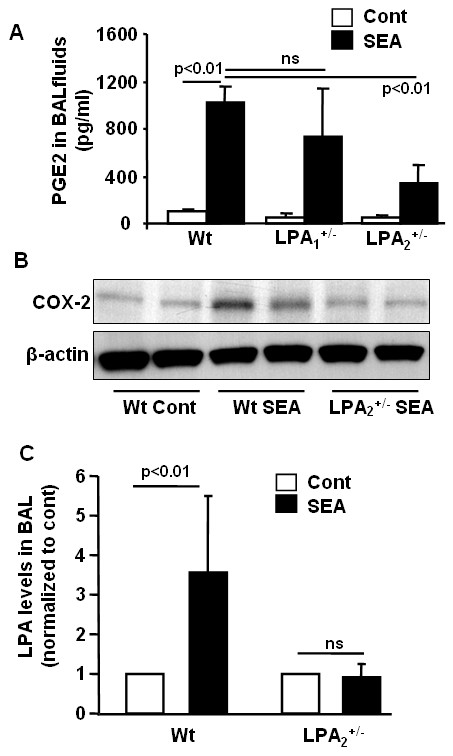
**LPA_2_^+/- ^mice exhibit a decrease in PGE2 and LPA levels in BAL fluids and COX-2 expression in lung tissue**. BAL fluids and lung tissue were collected from SEA unchallenged and challenged wild type, LPA_1_^+/-^, and LPA_2_^+/- ^mice (n = 4-6). **A) **PGE2 levels were measured by ELISA kit. **B) **Lung tissues were subjected to SDS/PAGE gel and COX-2 expression was determined by Western blotting. Representative image were shown. **C) **LPA levels in BAL fluids were quantified by LC-MS/MS and changes in LPA levels between wild type and SEA challenge mice were normalized to control levels.

Endogenous PGE2 is produced by airway epithelium, smooth muscle, dendritic cells, and macrophages in response to allergen challenge [35]. PGE2 has been shown to be an anti-inflammatory lipid mediator and bronchodilator in the airway [24,25]; however, administration of PGE2 induced various side effects, including cough, enhanced mucus production, and sensory nerve stimulation [36]. To determine the role of LPA receptors expression and PGE2 production in response to allergen challenge, we analyzed PGE2 levels in BAL fluids and COX-2 expression in lung tissues from *Schistosoma mansoni *eggs sensitized and challenged wild type mice. As shown in Fig. 5A, PGE2 levels were higher in control wild type and LPA_1_^+/- ^mice, compared to LPA_2_^+/- ^mice in response to *Schistosoma mansoni *eggs sensitization and challenge. *Schistosoma mansoni *eggs sensitization and challenge increased COX-2 expression in lung tissues of wild type mice while LPA_2_^+/- ^mice showed reduced COX-2 expression (Fig. 5B). Recently, we have shown that LPA induces COX-2 expression and PGE2 release in human bronchial epithelial cells [23]. As *Schistosoma mansoni *eggs sensitization and challenge increased LPA levels in BAL fluids (Table 2), we measured LPA levels in BAL fluids from sensitized and SEA challenged LPA_2_^+/- ^mice. Compared to wild type mice, LPA levels in BAL fluids from LPA_2_^+/- ^mice were decreased after SEA challenge. Together, these results suggest that increased lung COX-2 expression, PGE2 and LPA production in BAL fluids by *Schistosoma mansoni *eggs sensitization and challenge is regulated by LPA_2_.

### LPA_2 _deficiency on airway epithelial cells leads to reduced LPA mediated COX-2 expression and PGE2 release

**Figure 6:**
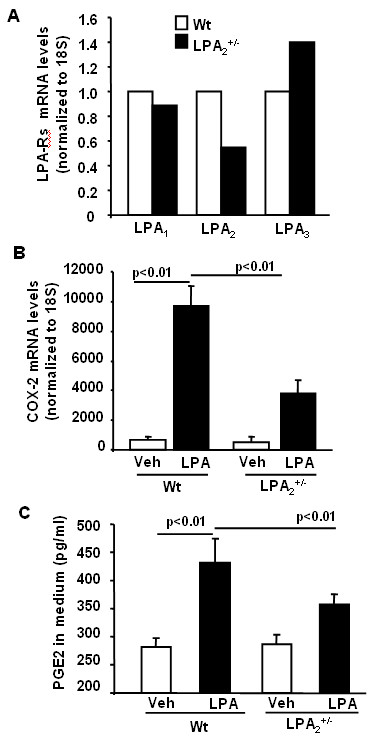
**LPA induces COX-2 expression and PGE2 release through LPA_2_**. Tracheal epithelial cells from wild type and LPA_2_^+/- ^mice were isolated as described in Materials and Methods and were cultured in 6-well plates. **A) **Total RNA was isolated and LPA receptors mRNA levels were measured by Real-time RT-PCR. **B) **Cells were challenged with 18:1LPA (1 μM) for 3 h, and COX-2 mRNA levels were measured by Real-time RT-PCR. **(C)**. Cells were challenged with 18:1LPA (1 μM) for 3 h, and medium were collected. PGE2 levels in medium were measured by ELISA kit.

Having demonstrated a role for LPA_2 _in *Schistosoma. mansoni *eggs-induced COX-2 expression, PGE2 secretion and airway inflammation, we hypothesized that expression of LPA_2 _on airway epithelial cells may be involved in inflammatory responses to *Schistosoma. mansoni *eggs sensitization and challenge. To investigate the role of LPA_2 _in LPA-induced COX-2 expression and PGE2 production, tracheal epithelial cells were isolated from wild type and LPA_2_^+/- ^mice. Analysis of total RNA for mRNA expression of LPA receptors by real-time RT-PCR revealed that expression of LPA_2_>LPA_4_>LPA_1 _≥ LPA_3 _in mouse tracheal epithelial cells (Table 3). In contrast to mouse tracheal epithelial cells, LPA_1 _and LPA_3 _were predominantly expressed in human bronchial epithelial cells [37]. In LPA_2_^+/- ^tracheal epithelial cells, expression of LPA_2 _mRNA was reduced to ~50%, compared to wild type mice, while there were no significant changes in expression levels of LPA_1 _and LPA_3 _mRNA (Fig. 6A). To determine the role of LPA_2_in LPA mediated COX-2 expression and PGE2 release, tracheal epithelial cells from wild type and LPA_2_^+/- ^mice were challenged with LPA (1 μM) for 3 h, total RNA isolated and COX-2 mRNA expression determined by Real-time RT-PCR. LPA stimulated COX-2 mRNA expression in wild type mouse cells (~13 fold); however, LPA-induced COX-2 mRNA expression was reduced in LPA_2_^+/- ^mouse cells (~56% of wild type cells) (Fig. 6B). The media, after LPA challenge, were collected and PGE2 levels were determined. As shown in Fig. 6C, PGE2 release from LPA_2_^+/-^mouse tracheal epithelial cells challenged with LPA was lower as compared to cells from wild type mice [PGE2 (pg/ml)-Wild type: vehicle, 268 ± 29; LPA, 432 ± 47; LPA_2_^+/-^: vehicle, 283 ± 21; LPA, 374 ± 16]. These results suggest that a role for LPA_2 _in LPA-induced COX-2 expression and PGE2 release from mouse tracheal epithelial cells.

**Table 3:** LPA receptors mRNA expression in lung tissue

LPA-Rs	LPA_1_	LPA_2_	LPA_3_	LPA_4_	LPA_5_
Normalized to 18S	49.5 ± 23.5	235.0 ± 15.5	46.0 ± 21.2	73.5 ± 6.7	n.d.

Total RNA were isolated from wild type mouse trachial epithelial cells and LPA receptors mRNA levels were determined by real-time RT-PCR with primers designed based on mouse LPA receptors mRNA sequence.

## Discussion

In the present study, we present several novel findings regarding LPA receptors expression, and its role in infiltration of eosinophils and lung inflammation in *Schistosoma mansoni *eggs sensitized and challenged murine model of asthma. We provide direct evidence for increased LPA levels in BAL fluids from *Schistosoma mansoni *eggs sensitized and challenged mice compared to control mice and a direct link between LPA_2_expression and lung inflammation mediated by *Schistosoma mansoni *eggs sensitization and challenge. The pro-inflammatory role of LPA_2 _is also evident from reduced PGE2 levels in BAL fluids and COX-2 expression in lung tissues of LPA_2_^+/- ^mice sensitized and challenged with *Schistosoma mansoni *eggs compared to controls. We also demonstrate that airway epithelial cells isolated from LPA_2_^+/- ^mice, compared to cells from wild type mice, exhibited reduced COX-2 expression and PGE2 release in response to LPA. To the best of our knowledge, this is the first report demonstrating a functional link between LPA, LPA_2 _and lung inflammation in a murine model of asthma.

Asthma is a Th2-type immune disease of the lung that is characterized by chronic inflammation, infiltration of inflammatory cells, reversible obstruction of airway hyperresponsiveness, mucus hypersecretion by goblet cells and remodeling of the bronchoalveolar structures. Th1 and Th2 cytokines play a key role in orchestrating inflammatory and structural changes of the airway in asthma by recruiting, activating and promoting inflammatory cells into the airway [38-40]. In addition to cytokines, lipid mediators such as prostaglandins, leukotrienes, platelet-activating factor, and lysophospholipids regulate immune and inflammatory responses in asthma [41-43]. Many of these lipid mediators exert their biological responses via GPCRs. Increasing sphingosine-1-phosphate (S1P) levels in circulation offers protection against lung injury in mice and S1P-receptor 1 (S1P_1_) heterozygous mice showed enhanced inflammation after LPS challenge suggesting an anti-inflammatory role of S1P_1 _[44]. The present study demonstrates the role of LPA and LPA_2_, a GPCR, in the pathogenesis of allergic airway inflammation in *Schistosoma mansoni *eggs sensitized and challenged murine model of asthma. LPA_1_^-/- ^mice generated from LPA_1_^+/- ^colonies, as compared to LPA_2_^-/- ^from LPA_2_^+/-^, showed 50% neonatal lethality and impaired suckling, and therefore, we decided to use LPA_1_^+/- ^and LPA_2_^+/- ^mice to investigate role of LPA receptors in airway inflammation. Although LPA_1_^+/- ^and LPA_2_^+/- ^mice exhibited less neutrophils infiltration, compared to wild type mice, after LPA challenge (Fig. 2), influx of eosinophils was lower in LPA_2_^+/-^, but not in LPA_1_^+/- ^mice after *Schistosoma mansoni *eggs sensitization and challenge (Fig. 3B). Both LPA_1_^+/- ^and LPA_2_^+/- ^mice showed reduced PAS positive cells in the bronchus compared to wild type after *Schistosoma mansoni *eggs sensitization and challenge (Fig. 4) suggesting the potential involvement of LPA_1 _and LPA_2 _in activation of goblet cells. These results indicate that activation of goblet cells are dependent on LPA_1 _and LPA_2_, however, only LPA_2 _is involved in chemotaxis of eosinophils into alveolar space after *Schistosoma mansoni *eggs sensitization and challenge. Our current results on infiltration of eosinophils in *Schistosoma mansoni *eggs sensitized and challenged murine model of asthma are in good agreement with increased numbers of eosiophils, a characteristic feature of human bronchial asthma, in biopsies of human lung tissues [40,45]. LPA is constitutively present in human BAL fluids and increased following allergic inflammation [28] and in patients with pulmonary fibrosis [29]. Intratracheal administration of LPA increased eosinophil influx in guinea pigs [22] and treatment of human eosinophils with LPA induced calcium mobilization, actin reorganization, and chemotaxis through Gαi-dependent LPA receptors [46]. In the present study, we found that LPA levels were increased by ~3 fold following *Schistosoma mansoni *eggs sensitization and challenge of wild type, which supports the notion of LPA as a chemotaxis factor of inflammatory cells in allergic inflammation.

The source of LPA accumulation and mechanism(s) of LPA generation in the lung after allergic inflammation is unclear. Our previous studies have demonstrated that acyl glycerol kinase (AGK) converts monoacylglycerol to LPA in human bronchial epithelial cells [47]. Further, phospholipase D (PLD) can also contribute to intracellular LPA generation by providing phosphatidic acid, a substrate for PA specific phospholipase A2 [48,49]. Interestingly, we observed that LPA levels in LPA_2_^+/- ^mice were significantly lower compared to wild type mice after *Schistosoma mansoni *eggs sensitization and challenge suggesting involvement of LPA_2_ and potentially other LPA receptors in regulation of LPA generation in the airway. The relative contributions of AGK and/or PLD pathways in LPA generation in response to *Schistosoma mansoni *eggs sensitization and challenge are unknown Additionally, extracellular LPA can be generated by lysoPLD (autotaxin), which converts lysophosphatidylcholine (LPC) to LPA [50]. Not only LPC levels were increased in BAL fluids of segmental allergen challenged patients [51], there was an increase in lysoPLD expression in LPS-stimulated monocytes [52], and stimulation of lysoPLD activity in asthmatic patients [53]. Thus, increase in LPC levels and lysoPLD expression and activity may be involved in enhanced LPA generation during lung inflammation. Further studies are needed to establish the potential source(s) of LPA in BAL fluids and mechanism(s) of LPA generation during allergic lung inflammation.

In contrast to LPA, there are only a few reports that describe the role of LPA receptors in lung inflammation, injury and remodeling. Deletion of LPA_1 _reduced fibroblast recruitment and vascular leak in the bleomycin model of pulmonary fibrosis [29] while LPA/LPA_2 _signaling via αvβ6 integrin-mediated activation of TGF-β has been implicated in the development of bleomycin-induced lung fibrosis in mice [54]. Down-regulation of LPA_2 _by siRNA attenuated LPA-induced phosphorylation of p38 MAPK/JNK, and IL-8 secretion in human bronchial epithelial cells [37]. Interestingly, *Schistosoma mansoni *eggs sensitization and challenge induced COX-2 expression and PGE2 was significantly attenuated in LPA_2_^+/-^, but not LPA_1_^+/-^, mice suggesting a potential link between reduced LPA_2 _expression and COX2/PGE2 levels. In accordance with our *in vivo *results on *Schistosoma mansoni *eggs mediated COX-2 expression and PGE2 release in mouse lungs, tracheal epithelial cells from LPA_2_^+/- ^mice exhibited decreased COX-2 expression and PGE2 release in response to LPA as compared to cells from wild type mice. Further, our results with LPA_2_^+/- ^mice suggest a role for LPA_2 _in the influx of eosinophils and lung inflammation induced by *Schistosoma mansoni *eggs sensitization and challenge suggest a role for LPA signaling via LPA_2 _in pro-inflammatory responses.

## Conclusion

The present study demonstrates increased LPA levels in BAL fluids in a murine model of asthma and LPA_2 _heterozygous knockout mice show reduced Th-2 dominant airway inflammatory responses. These results suggest that endogenous LPA and LPA_2 _play a critical role in pathogenesis of airway inflammatory diseases. Therapeutic targeting of LPA_2 _may be beneficial in reducing allergic inflammatory responses in airway diseases.

## Competing interests

The authors declare that they have no competing interests.

## Authors' contributions

The study was designed and the protocol developed by YZ, JT, AIS, and VN. DH and SP carried out the genotyping. BE carried out the LPA measurement. JC provided the LPA_1 _and LPA_2 _heterozygous mice. All the authors read and approved the final manuscript.
